# Effect of Deposition Parameters on Electrochemical Properties of Polypyrrole-Graphene Oxide Films

**DOI:** 10.3390/ma13030624

**Published:** 2020-01-31

**Authors:** Alina Iuliana Pruna, Nelly Ma. Rosas-Laverde, David Busquets Mataix

**Affiliations:** 1 Center for Surface Science and Nanotechnology, Polytechnic University of Bucharest, 060042 Bucharest, Romania; 2 Institute of Materials Technology, Universitat Politècnica de València, 46022 Valencia, Spain; dbusquets@mcm.upv.es; 3 Department of Materials, Escuela Politécnica Nacional, Quito 170524, Ecuador; nelly.rosas@epn.edu.ec; 4 Department of Materials and Mechanical Engineering, Universitat Politècnica de València, 46022 Valencia, Spain

**Keywords:** graphene oxide, polypyrrole, electrodeposition, capacitance

## Abstract

Graphene oxide (GO)-modified polypyrrole (PPy) coatings were obtained by electrochemical methods in the presence of the anionic surfactant, sodium dodecyl sulfate (SDS). The structure, morphology, and electrochemical properties of the coatings were assessed by Fourier transform infrared (FTIR) spectroscopy, Raman spectroscopy, scanning electron microscopy (SEM) and cyclic voltammetry at varying scan rates, respectively. The properties of the obtained coatings were analyzed with the GO and PPy loadings and electrodeposition mode. The hybrid coatings obtained galvanostatically showed a coarser appearance than those deposited by cyclic voltammetry CV mode and improved performance, respectively, which was further enhanced by GO and PPy loading. The capacitance enhancement can be attributed to the SDS surfactant that well dispersed the GO sheets, thus allowing the use of lower GO content for improved contribution, while the choice of suitable electrodeposition parameters is highly important for improving the applicability of GO-modified PPy coatings in energy storage applications.

## 1. Introduction

The long cycle life, high power and energy density, and short-time charge/discharge of the electrochemical capacitors allow them to fill the technology gap between batteries and traditional capacitors [1,2,3,4,5,6]. Electrochemical capacitors have a wide range of applications including electronic vehicles, mobile communications, and back-up systems [2,6,7,8,9]. Two mechanisms are involved in the energy storage: (i) electrical double layer capacitance due to adsorption/desorption processes at the electrode/electrolyte interface for which carbon based materials including carbon nanotubes, graphene, and activated carbon are considered due to their high specific surface area and long cycle life; and (ii) pseudocapacitance, which involves rapid and reversible surface Faraday reactions and usually employs a transition metal oxide/hydroxide or conducting polymers due to their high specific capacitance and energy density [2,4,6,8,10].

The combination of materials such as conducting polymers with carbon nanomaterials has attracted increased interest in the last years in order to overcome the drawbacks these materials have as a singular component and improve their properties by synthesizing a hybrid material with a synergetic effect on properties including high energy and power density, cyclability [5], and stability [11]. For example, polypyrrole (PPy) is one of the most used conjugated polymers [10] in energy storage application [4], thanks to desirable properties including high electrical conductivity, good redox properties, good environmental stability and aqueous solubility, low density, non-toxicity, low cost, and the ease of synthesis [12,13,14]. PPy is highly employed in varying fields including electrochemical sensing, drug delivery systems [12,15], and supercapacitors, thanks to its high specific capacitance [7] (theoretical value of 620 F·g^−1^ [16]). PPy has two drawbacks: on one hand, the slow diffusion of electrolyte ions within its matrix during the redox process [10], which results in poor power capability [9]; and on the other, the volume variation as a consequence of the ion doping/dedoping [9] during the charge/discharge process, which results in poor stability for long-term cycling [6,16]. On the other hand, graphene, a two-dimensional single layer of carbon with a sp^2^ hybridization [1,17] shows zero band gap [17], high electrical conductivity (106 S·cm^−1^ [10]), high specific surface area (~2600 m^2^·g^−1^), great mechanical properties [5,7] and chemical stability [6], and it has attracted significant research for use in supercapacitors, solar cells, and electrochemical sensors [1]. Graphene has drawbacks in its limited capacitance values as well as low applicability in hybrids [6]. In this respect, a derivative of graphene, graphene oxide (GO), can be employed as it possesses oxygen groups obtained by varying methods including wet chemistry, which confer it with high dispersibility in varying solvents [17]. 

The research on the synergetic effects of GO and PPy has shown that GO can be used as a weak electrolyte support and charge-balancing dopant for PPy [13,16,18,19] as well as mechanical support in order to increase the porosity of the PPy and solve drawbacks related to the damage of PPy during the charge/discharge process [16,19]. On the other hand, PPy can be employed as a matrix in order to overcome the drawbacks of GO nanomaterials such as the low electrochemical properties attributed to its electric double-layer capacitors (ELDC) behavior and the hydrophobic nature of its reduced form [3]. Thus, the π–π interaction between the GO and PPy can facilitate the charge transport, which would improve the electrical conductivity and electrochemical performance of the obtained hybrid [2,7,20].

There are many methods including chemical and electrochemical deposition, which can be applied to synthesize PPy/rGO hybrids [16]. Amongst such methods, electrochemical deposition is an easy, cheap, rapid, and simple method [21] which allows the fabrication of PPy/rGO composites without the necessity of other chemical binders [22]. Moreover, electrodeposition allows a high degree of control on the morphology, aspect ratio, and performance of coatings by simply varying the deposition conditions including deposition cycles [21], pH, potential, and temperature [11], so that good electrical connection and low internal resistance coatings are achieved [18]. The marked influence of GO on the morphology of the PPy/rGO hybrids with respect to its concentration has been reported as the nucleation sites for PPy growth are adjusted [16]. 

The loading of active material can be controlled by electrodeposition parameters such as deposition duration [6], deposition charge density [20], the applied potential or precursor concentration [13]. The electrodeposition modes for the synthesis of PPy/rGO hybrids usually include cyclic voltammetry (CV), potentiostatic, and galvanostatic modes [21]. Specifically, CV mode allows the synthesis of a PPY/rGO hybrid in a 1-step procedure by either incorporating GO sheets into the polymeric matrix during electropolymerization, or by the reduction of GO at negative potential sweeping while PPy is electropolymerized by sweeping the potential in the anodic direction [22]. The areal capacitance has been reported to improve with the increase of deposition time at low scan rates [6,16]. For instance, Zhou et al. obtained the maximum areal capacitance of 152 mF·cm^−2^ at 10 mV·s^−1^ for a PPy/GO composite obtained galvanostatically at 1.0 mA cm^-2^ from a 0.25 M pyrrole monomer and 2 mg·mL^−1^ GO [6], while Cao et al. obtained a maximum of 387.6 mF·cm^−2^ at 0.2 mA·s^−1^ for a PPy/GO composite obtained potentiostatically at 0.8 V from 0.072 M pyrrole aqueous solution containing 0.1 mg·mL^−1^ GO [16].

While many improvements have been reported, the effect of the electrochemical deposition parameters on the properties of a PPy/rGO hybrid still lacks understanding, especially on co-electrodeposited hybrids. Cao et at. reported the performance of PPy/GO electrodes was strongly influenced by the GO loading and suggested that 0.4 mg·mL^−1^ is the critical concentration for improving the performance with deposition duration [16]. On the other hand, Halder et al. reported that the use of an anionic surfactant could be employed to interact with the carboxyl groups of GO and separate layers of GO to exhibit improved performance [23].

This work presents a systematic study on the most important parameters affecting the properties of the hybrid: GO content, electrodeposition mode, loading of active material, and type of GO incorporation into the hybrid, that is, on one hand, the incorporation during electropolymerization and on the other, a layer by layer deposition of the components. In this work, an anionic surfactant (sodium dodecyl sulphate, SDS) was employed in order to obtain highly active well separated layers of GO that would allow the use of low GO content for boosting polypyrrole properties. The obtained results show that the electrochemical properties of PPy/rGO can be finely adjusted by controlling the electrochemical deposition parameters, thus proving with useful knowledge for the application of such hybrids in various emerging fields. 

## 2. Materials and Methods 

### 2.1. Materials

The chemicals were reagent grade (Sigma Aldrich, Saint Louis, MO, USA) and used as received, except for pyrrole, which was distilled prior to use. All electrolytes were obtained with distilled water. Carbon fiber papers (TGP-H-060, Fuel Cell Store) were employed as the substrate. Prior to use, the substrates were cleaned in acetone in an ultrasonic bath for 10 min and then dried in air. For PPy deposition, the electrolyte consisted of an aqueous solution of 0.05 M sodium p-toluenesulfonate (NapTS) containing 0.1 M Py and 20 mM sodium dodecyl sulfate (SDS). Although GO could serve as a supporting electrolyte for the deposition of PPy [24], and its oxygen groups would strongly interact with nitrogen atoms in the NH group of the PPy back-bone [25], the NapTS was employed to improve the conductivity of the solution. The anionic surfactant (SDS) has a two-fold role in that not only does it improve the dispersion and uniformity of the final coating and help the wetting of the substrate, but it can also help in better separation of the GO sheets, which could improve their interaction with the polymeric matrix and thus reduce the required GO amount.

### 2.2. Synthesis of PPY/rGO Hybrid Coatings by CV Mode

The substrate modification with the PPy/rGO hybrid coating by CV was studied with the type of GO incorporation into the hybrid. Thus, 1-step deposition based on the incorporation of GO in the PPy matrix during the electropolymerization of pyrrole exploited the use of GO as the dopant and surfactant. On the other hand, a 2-step deposition consisting of layer by layer deposition of GO and PPy was applied in order to enhance the role of GO in the nucleation and growth of PPy. 

The deposition by CV was performed by sweeping the potential in the 0 to 1 V range for a varying number of cycles between 10 and 70. In the 1-step deposition of the PPy/rGO hybrid coating, a varying GO content from 0.25 mg·L^−1^ to 1 mg·L^−1^ was added to the electrolytic bath and subjected to ultrasonic homogenization for 30 min. In the 2-step deposition, the substrate was first activated as reported elsewhere [26] in order to improve its hydrophilicity. Then, the substrates were modified with GO nanomaterial by dip coating in a 0.5 mg·mL^−1^ GO solution previously sonicated for 30 min. Three immersion cycles for different durations were employed in order to analyze the effect of GO loading, namely 1 min and 30 min immersion in an ultrasonic bath, followed by rinsing with distilled water, and drying in airflow heated at 80 °C. The GO coating was further reduced to rGO by the CV method in 0.1 M KCl solution by sweeping the potential in the 0 to −1.4 V range at 50 mV·s^−1^ for 10 cycles. Finally, the second layer, polypyrrole, was deposited by CV in the same conditions as previous, at the surface of the rGO coating. 

### 2.3. Synthesis of PPy/rGO Hybrid Coating by Galvanostatic Mode

In order to observe the effects of GO sheets on the electropolymerization of pyrrole, galvanostatic deposition of the hybrid coating was also achieved in a 1-step deposition. The substrate modification with the PPy/rGO hybrid coating was performed by applying a constant current density of 1 mA·cm^−2^ for varying durations from 120 s to 1200 s from a solution of 0.05 M sodium p-toluenesulfonate (NapTS) containing 0.1 M Py and 20 mM sodium dodecyl sulfate (SDS), and varying GO content from 0.25 mg·L^−1^ to 1 mg·L^−1^.

### 2.4. Characterization

All the electrochemical depositions were carried out by using a potensiostat Princeton Applied Research Versastat 3 in a conventional three-electrode electrochemical cell where the working electrode was the carbon fiber substrate. A Pt foil was employed as the counter-electrode and the reference electrode was Ag/AgCl in saturated KCl. The obtained films were characterized in terms of structural and morphological characteristics by Fourier transform infrared spectroscopy (FTIR, Spectrum 100, Perkin Elmer, Waltham, MA, USA), Raman spectroscopy (inVia (Renishaw, Wotton-under-Edge, UK) microscope employing a 514-nm laser), and scanning electron microscopy (Jeol, working at 20 kV). The electrochemical analysis of the 2-step electrodeposited PPy/rGO coating was performed in a 0.5 M Na_2_SO_4_ solution in a potential range from −0.1 to 0.7 V at varying scan rates between 2 and 100 mV·s^−1^. The electrochemical analysis of 1-step PPy/rGO was performed in a 0.5 M Na_2_SO_4_ solution by CV at varying scan rates between 2 and 100 mV·s^−1^.

## 3. Results

**Figure 1:**
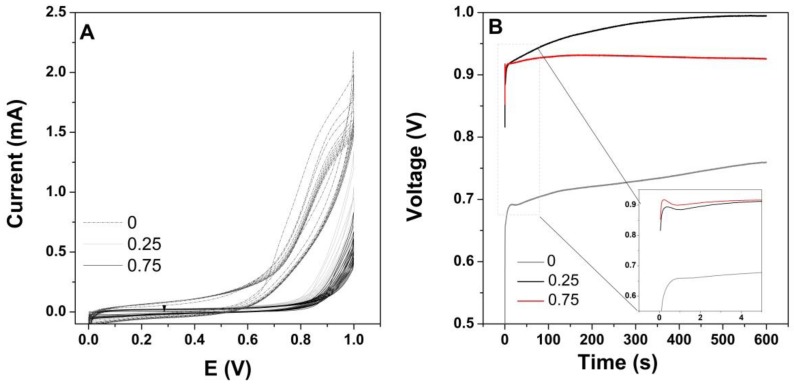
One-step deposition of polypyrrole (PPy) with graphene oxide (GO) content (mg·L^−1^) by: (**A**) cyclic voltammetry and (**B**) galvanostatic mode.

The effect of GO as the dopant was studied by adding the GO nanomaterial to the electrolytic bath and subjecting the carbon fiber substrate to a 1-step modification by both CV mode and constant current mode. The CVs corresponding to the electrodeposition of the PPy coating, before and after doping with the GO nanomaterial, is presented in Figure 1A. The electropolymerization onset in the case of the pure PPy appeared at about 0.6 V, while in the presence of GO, it shifted toward 0.8 V. The deposition current increased with the cycle number, however, it decreased with the addition of GO and its content extent. The 1-step co-electrodeposition by galvanostatic mode is shown in Figure 1B, depicting the chronopotentiometric curves as a function of GO loading. Upon the initial voltage increment [20] attributed to the nucleation stage, a potential plateau was achieved. As can be seen, the galvanostatic co-electrodeposition of GO and PPy required increased voltage with the addition of GO, which is in agreement with the CV results, however, there was little difference with an increase in GO loading.

**Figure 2:**
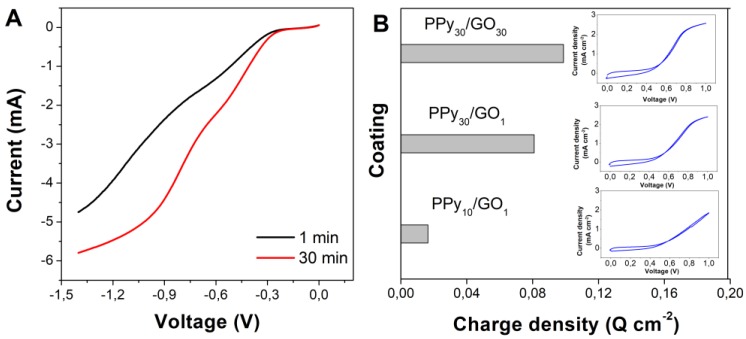
(**A**) Effect of immersion time on the reduction of GO and (**B**) the effect of PPy deposition cycle number and GO loading (immersion time) on the PPy deposition charge.

In the 2-step CV deposition, the GO deposited as the first layer needs to be partially reduced, thus it was subjected to electrochemical reduction by sweeping the potential up to −1.4 V. As can be seen in Figure 2A, which shows the cathodic scan evolution, a higher current was obtained with the increase in GO loading, along with a shift in the reduction potentials. The evolution of the PPy deposition charge with varying GO loading and PPy cycle number is further depicted in Figure 2B. The increase in material loading resulted in an obvious increase in PPy deposition charge as indicated by the electropolymerization onsets and current depicted in the cyclic voltammograms in Figure 2B. 

**Figure 3:**
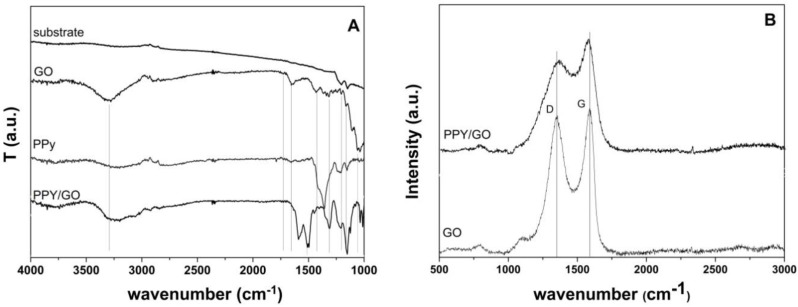
(**A**) Fourier-transform infrared spectroscopy FTIR spectra of the PPy, GO, and the PPy/GO hybrid at the surface of the carbon fiber paper substrate; (**B**) the Raman spectra of the GO and PPy/GO hybrid at the surface of the carbon fiber paper substrate.

The formation of the PPy–GO composite onto carbon fiber substrate was inspected by FTIR and Raman spectroscopy. The typical FTIR spectra of single components and the hybrid are depicted in Figure 3A. The spectra of GO exhibits typical peaks assigned to carbonyl/carboxyl C=O, aromatic C=C, epoxy C–O, and alkoxy C–O, which are located at about 1720, 1640, 1426, 1210, 1152 cm^−1^, and 1058 cm^−1^ [27,28,29,30,31,32], and a large band between 3000 and 3500 cm^−1^ was attributed to adsorbed water molecules. The electropolymerized pyrrole showed typical FTIR peaks attributed to the N–H in-plane deformation vibration located at 1043 cm^−1^ and C=C vibrations in the PPy rings located at 1652 cm^−1^ [33,34]. The PPy, upon the addition of GO, aside from showing the peaks of single components also showed a peak at 1503 cm^−1^, which was ascribed to C–N stretching in the pyrrole ring and a wide band at 3230 cm^−1^ was attributed to the N–H stretching vibration [35]. No significant differences were observed in the FTIR spectra of PPy hybridized with GO by different deposition modes.

The Raman spectra are further depicted in Figure 3B. The typical D and G bands assigned to disordered carbon vibrations and sp^2^ hybridized carbons, respectively, were identified in the spectrum of GO with locations at 1352 cm^−1^ and 1588 cm^−1^ [36]. Upon its addition to the electrolytic bath, the obtained GO-modified PPy coating showed similar features due to an overlap of the bands, however, it can be clearly seen that the G band slightly shifted to 1570 cm^−1^. Moreover, the D to G intensity ratio decreased upon the hybridization of PPy with GO nanomaterial.

**Figure 4:**
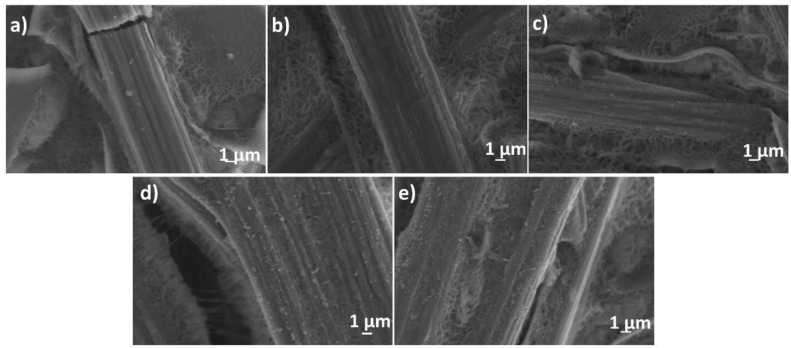
Scanning electron microscopy (SEM) images of carbon fiber before (**a**) and after coating with PPy by cyclic voltammetry (CV) (**b**) and galvanostatically (**c**), and with PPy deposited galvanostatically in the presence of 0.25 and 0.75 mg·L^−1^ GO (**d**,**e**), respectively.

The surface morphology of the carbon fiber substrates coated with the PPy and PPy-GO hybrid coating was analyzed by SEM as shown in Figure 4. The presence of the PPy coating can be clearly assessed by comparing the image of carbon fibers before (Figure 4a) and after the electro-coating process (Figure 4b–e), where the nodular-like morphology of PPy covers the carbon fiber, resulting in a larger diameter. The PPy deposited by CV mode exhibited a smooth appearance, following the morphology of the substrate closely, while the PPy deposited galvanostatically appeared rougher with respect to the coating deposited by CV. The addition of GO resulted in the appearance of flakes and an increased number of nodules, as indicated in Figure 4d,e depicting the carbon fiber coated with PPy hybridized with an increasing content of GO.

**Figure 5:**
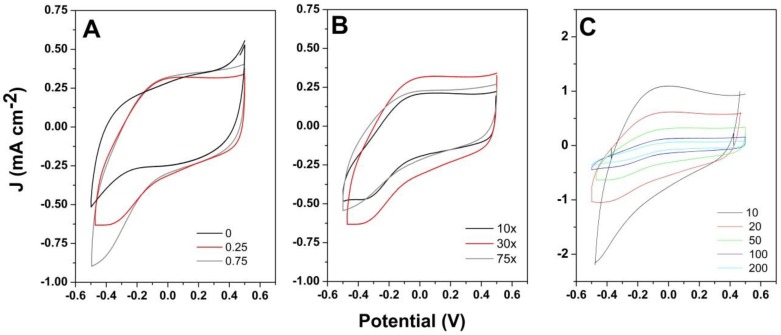
CV curves at 50 mV·s^−1^ of (**A**) PPy/rGO obtained by CV mode for 30 cycles as a function of GO content; (**B**) PPy obtained by CV with addition of 0.25 mg·L^−1^ GO for varying cycle number; (**C**) CVs of PPy obtained by CV for 30 cycles with addition of 0.25 mg·L^−1^ GO as a function of scan rate (mV·s^−1^).

The electrochemical properties were evaluated by cyclic voltammetry in a large potential window from −0.5 to 0.5 V at varying scan rates. Figure 5A shows the electrochemical properties of the PPy/rGO electrode obtained by 1-step CV mode as a function of GO content. It can be observed that a pair of cathodic and anodic peaks at about −0.37 V and −0.07 V, respectively, appeared when GO was added as the dopant and increased with GO content. The shape of the voltammogram changed from a quasi-rectangular one with an increase in GO content. The increase in GO-doped PPy deposition cycles up to 30 was observed to produce an enhancement of the current signal after which it decreased, reaching a similar response to a deposition for 10 cycles when deposition was performed for 75 cycles. The scan rate increase resulted in higher CV response, which indicates a lower areal capacitance.

**Figure 6:**
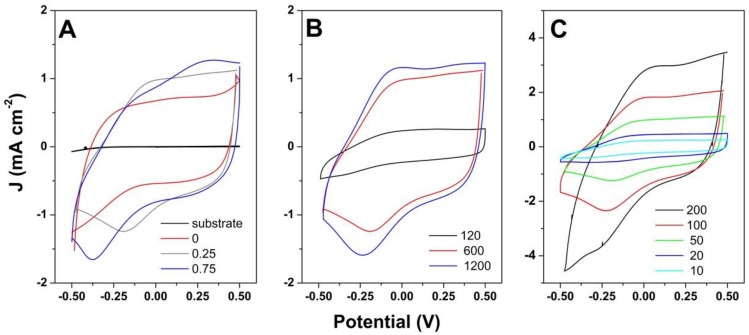
CV curves at 50 mV·s^−1^ of (**A**) PPy/rGO obtained by galvanostatic mode for 600 s as a function of GO content (mg·L^−1^); (**B**) PPy hybridized with 0.25 mg·L^−1^ GO by galvanostatic mode at 1 mA cm^−2^ for varying duration (s); (**C**) CVs as a function of scan rate (mV·s^−1^) for PPy hybridized with 0.25 mg·L^−1^ GO at 1 mA·cm^−2^ for 600 s.

The effect of GO as the dopant in a 1-step deposition by galvanostatic mode in varying conditions is further presented. Figure 6A depicts the effect of GO loading on the electrochemical response of hybrid coating upon cycling at 50 mV·s^−1^. It can be observed that the addition of GO resulted in increased current response and a pair of redox peaks appeared at −0.06 and −0.19 V, which shifted with the increase in GO loading. Moreover, another peak located at about 0.35 V appeared at the highest GO content. The electrochemical behavior of the hybrid coating deposited galvanostatically at 1 mA·cm^−2^ for varying duration, depicted in Figure 6B, showed that the deposition duration resulted in an increase in current response, reaching the highest value at 1200 s. The pair of redox peaks observed previously slightly shifted to more negative values with deposition duration. The evolution of current response with scan rate for a galvanostatically deposited hybrid coating (shown for 600 s deposition duration) in Figure 6C shows an increase in current, which results in a lower areal capacitance with scan rate.

For comparison purposes, the effect of GO as a buffer layer for PPy nucleation and growth was studied in a 2-step procedure involving the immersion of the substrate in a GO dispersion and its reduction by electrochemical means, followed by the deposition of PPy by CV mode. It was observed (not shown) that the CVs exhibited a similar shape with the previous samples, however, no peaks were identified. 

**Figure 7:**
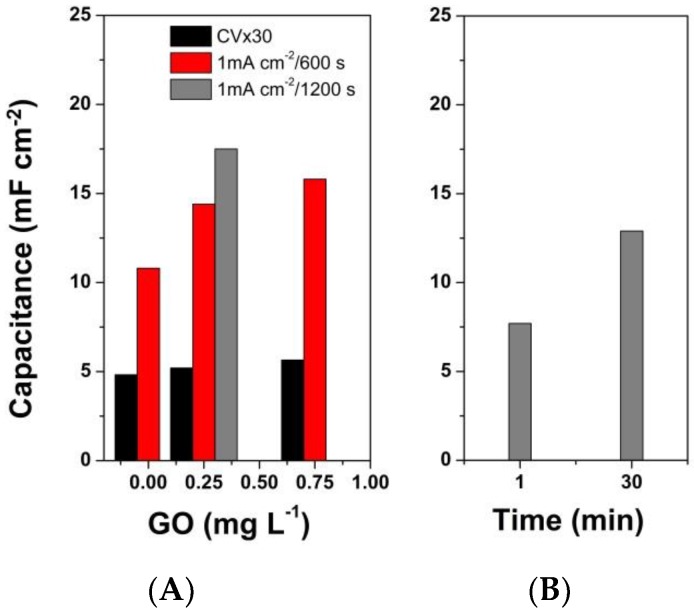
Specific capacitance of the PPy/rGO hybrids deposited in: (**A**) 1-step procedure as a function of GO content and (**B**) 2-step procedure as a function of immersion duration in GO dispersion.

The CV results obtained at 50 mV·s^−1^ were converted to specific capacitance data and the evolution of the capacitance with the procedure (1-step by CV or galvanostatic and 2-step by CV) is depicted in Figure 7. It can be observed that the capacitance improved with GO loading by the 1-step electrodeposition mode, however, galvanostatic deposition appeared to result in enhanced capacitance with respect to the CV one. The increased deposition time by galvanostatic mode even for a low GO content of 0.25 mg·L^−1^ resulted in an improved capacitance value. On the other hand, the increased GO loading by duration of immersion in the layer by layer deposition appears to have a similar effect on the capacitance.

## 4. Discussion

During the co-electrodeposition process, the PPy monomer and GO sheets are attracted by the π–π and electrostatic interactions [37]. As observed in Figure 1A, the electropolymerization onset corresponding to the oxidation of Py monomer [38] shifted from 0.6 V to 0.8 V with the addition of GO as the dopant in a 1-step deposition of the PPy/rGO hybrid, which is due to the insulating nature of GO as many oxygen groups are decorating its surface. The lower current obtained in the presence of GO was due to its low conductivity [24]. As the area of the cyclo voltammogram indicates the loading with active material [11], it was observed that the coatings contained less active material upon the addition of GO by CV deposition. The galvanostatic co-electrodeposition results confirmed the increased voltage necessary for deposition when GO is added. The increase in GO loading appeared to result in faster nucleation, which is indicative of the interaction between the GO and PPy.

On the other hand, in the 2-step cyclic voltammetry deposition, the GO is exploited for the nucleation of PPy as a conductive layer [33], thus its decorating oxygen groups need to be removed, as indicated by the reduction peaks in the cathodic scans of the GO electrode in Figure 2A located at about −0.7 V and −1.3 V, and attributed to the reduction of varying oxygen groups in GO. The increase in GO loading exposes more oxygen groups to the reduction process, as indicated by the increase in cathodic current and the shift toward more anodic values of reduction potentials (−0.55 V and −1.1 V). The effect of GO loading and cycle number for the deposition of PPy was further investigated. By triplicating the cycle number, the PPy deposition current increased, as observed in the last deposition cycle in Figure 2B, which resulted in a larger deposition charge. The extent of the increase in deposition charge was higher than expected, which indicates the marked participation of residual oxygen groups in reduced GO for the nucleation of PPy. On the other hand, a current flattening occurs upon cycling, which indicates a transport limitation in the PPy film [39]. The increase in GO loading appears to only slightly increase the deposition charge of PPy, which can be attributed to the lower oxygen groups in the reduced GO.

The hybridization of PPy with GO by either procedure was studied by FTIR and Raman spectroscopy. The FTIR spectra of the hybrid coatings in Figure 3A show the typical bands of the single components along with a more intense peak at 1503 cm^−1^, attributed to the C–N stretching in the pyrrole ring [35]. The Raman spectra in Figure 3B show an overlap of the typical bands of rGO and PPy and a shift in the G band, along with a lower D/G intensity ratio D/G, which are indicative of the hybridization of rGO and PPy and the charge transfer between the PPy and rGO [40]. The morphology analysis indicates that the PPy deposited galvanostatically appeared rougher with respect to the coating deposited by cyclic voltammetry, which can be attributed to the presence of GO flakes.

The cyclic voltammetry study of the hybrid coatings obtained by CV mode showed the current response improved with material loading (GO amount and deposition cycles). A pair of redox peaks appeared in Figure 5A and shifted with increased GO addition/coating loading (Figure 5B), which indicates the participation of GO to the electrochemical response by introducing active sites in the polymeric matrix. Similar results were obtained for the 1-step deposition by galvanostatic mode, where increased GO loading (Figure 6A) and deposition duration (Figure 6B) improved the current response, as demonstrated elsewhere [6,16]. Moreover, the GO addition generates higher specific surface area and the π–π interactions between GO and PPy [20] that enhance the electrochemical performance. With respect to CV deposited coatings, the galvanostatic ones exhibited more intense redox peaks, which indicated stronger interaction between rGO and PPy toward improved performance. At higher GO loading, another peak appeared, which was attributed to the insertion of Na^+^ ions from the electrolyte, as the negative charge introduced by GO needs to be balanced with positive ions from the electrolyte [24]. 

As the CV area was employed for the calculation of the capacitance of the electrode [20], the specific capacitance value was observed to decrease with the increase in scan rate, irrespective of the hybrid coating, which is due to the fact that only part of the active material participates in the charge/discharge process at high scan rates [6].

By comparing the capacitance evolution for the hybrid coatings, it was observed that CV deposition mode had limitations in improving the electrochemical performance, while the coatings deposited by galvanostatic mode could improve in performance by increasing GO loading or by increasing deposition duration (Figure 7A), which can be attributed to enhanced incorporation of GO flakes into the polymeric matrix during co-electrodeposition. Figure 7B shows the coatings deposited by the 2-step mode could improve their performance by tailoring the GO loading and the interaction between the PPy and residual oxygen groups of rGO as a result of the electrochemical reduction of GO. 

The obtained results demonstrate that the capacitance properties of GO-modified PPy coatings could be easily improved without it being necessary to employ other nanoparticles, complex architectures, or harmful reagents [6,16,33,41,42,43]. Despite the fact that the varying test parameters allowed only a qualitative comparison with the literature, it was observed that the performance of the obtained thin hybrid PPy/rGO coatings was similar to other reports [41]. The benefits of the present work consist of the improvement of the capacitance properties by simply adjusting the electrochemical parameters while using less added GO, thanks to the use of an anionic surfactant to better disperse the GO sheets and enhance their reactivity. Therefore, the results obtained in this work could provide useful knowledge for the synthesis of electrode materials with improved performance.

## 5. Conclusions

Polypyrrole was hybridized with a low content of GO layers by 1-step co-electrodeposition by CV and galvanostatic modes. Two-step electrodeposition of hybrid coatings was employed for comparison. An anionic surfactant, SDS, was employed to well separate the GO sheets in order to exploit the contribution of GO to the properties of the hybrid coatings. The effect of GO loading and electrochemical parameters such as deposition cycles and deposition duration were investigated. The obtained coatings were characterized in terms of structure, morphology, and electrochemical properties. The hybridization was confirmed by both the FTIR and Raman spectroscopy results in all cases, however stronger interaction between PPy and GO was indicated for the galvanostatic deposition mode. The morphology analysis indicated coarser coatings with a higher surface area by galvanostatic deposition mode in comparison to cyclic voltammetry. The electrochemical results showed that the performance of the hybrid coatings improved with the GO and PPy loading. The results reported in this work indicate that anionic surfactants could be exploited for tuning the electrochemical properties of GO-modified polypyrrole coatings by allowing the use of lower GO content while the adjustment of electrodeposition parameters is highly important for improving the applicability of such-modified PPy coatings toward energy storage applications.
